# Maternal Undernutrition Significantly Impacts Ovarian Follicle Number and Increases Ovarian Oxidative Stress in Adult Rat Offspring

**DOI:** 10.1371/journal.pone.0015558

**Published:** 2010-12-13

**Authors:** Angelica B. Bernal, Mark H. Vickers, Mark B. Hampton, Rebecca A. Poynton, Deborah M. Sloboda

**Affiliations:** 1 The Liggins Institute, The University of Auckland and the National Research Centre for Growth and Development, Auckland, New Zealand; 2 Free Radical Research Group, Department of Pathology, University of Otago, Christchurch, New Zealand; Indiana University, United States of America

## Abstract

**Background:**

We have shown recently that maternal undernutrition (UN) advanced female pubertal onset in a manner that is dependent upon the timing of UN. The long-term consequence of this accelerated puberty on ovarian function is unknown. Recent findings suggest that oxidative stress may be one mechanism whereby early life events impact on later physiological functioning. Therefore, using an established rodent model of maternal UN at critical windows of development, we examined maternal UN-induced changes in offspring ovarian function and determined whether these changes were underpinned by ovarian oxidative stress.

**Methodology/Principal Findings:**

Our study is the first to show that maternal UN significantly reduced primordial and secondary follicle number in offspring in a manner that was dependent upon the timing of maternal UN. Specifically, a reduction in these early stage follicles was observed in offspring born to mothers undernourished throughout both pregnancy and lactation. Additionally, antral follicle number was reduced in offspring born to all mothers that were UN regardless of whether the period of UN was restricted to pregnancy or lactation or both. These reductions were associated with decreased mRNA levels of genes critical for follicle maturation and ovulation. Increased ovarian protein carbonyls were observed in offspring born to mothers UN during pregnancy and/or lactation and this was associated with peroxiredoxin 3 hyperoxidation and reduced mRNA levels; suggesting compromised antioxidant defence. This was not observed in offspring of mothers UN during lactation alone.

**Conclusions:**

We propose that maternal UN, particularly at a time-point that includes pregnancy, results in reduced offspring ovarian follicle numbers and mRNA levels of regulatory genes and may be mediated by increased ovarian oxidative stress coupled with a decreased ability to repair the resultant oxidative damage. Together these data are suggestive of maternal UN potentially contributing to premature ovarian ageing in offspring.

## Introduction

There is now considerable epidemiological and experimental evidence indicating that early life environmental signals, including nutrition, affect subsequent development leading to patho-physiologies including obesity and insulin resistance [1]. These signals induce highly integrated responses in endocrine-related homeostasis, resulting in persistent changes to the developmental trajectory producing an altered adult phenotype. This phenomenon has been termed developmental programming, whereby early life events trigger processes that prepare the individual for particular circumstances that are anticipated in the postnatal environment [1]–[3]. However, where the intrauterine and postnatal environments differ markedly, such modifications to the developmental trajectory may prove maladaptive in later life [3]. Evidence that reproductive maturation and function is similarly influenced by early life events is now emerging from animal studies and human populations [4], [5]. It is also clear that in many cases the prenatal and postnatal nutritional environments interact to influence reproductive function in offspring [4], [5].

Ovarian function is exquisitely sensitive to nutritional status and clinical and experimental studies have demonstrated that a decline in ovarian follicular reserve, changes in ovulation rates and altered age at onset of menarche are all vulnerable to early life influences [5], [6]. We have previously shown that nutritional deficits and excesses during intrauterine, lactational and post-weaning periods resulted in the acceleration of pubertal onset and subsequent changes in ovarian function [5]. The mechanisms underlying these changes however are unclear. Regulatory factors known to influence reproduction have become a focus of investigation and studies have demonstrated that, in addition to its role in energy metabolism, the adipokine leptin acts as a metabolic signal to the central reproductive axis [7]. Leptin has been reported to have both stimulatory and inhibitory actions on ovulatory processes and ovarian steroidogenesis [8], [9] and act as a permissive neuro-regulatory factor for the onset of puberty [10]–[12].

It has been suggested that altered oxidative stress conditions may underpin many of the known associations between early life nutritional adversity and altered later life physiologic function [13], [14]. In this regard, it has been shown that mitochondrial function was altered in embryos and oocytes of mothers that were nutritionally challenged [15]. It is well recognized that disruption of redox homeostasis alters gene expression and that the accumulation of oxidized proteins has a deleterious effect on cell function [16], [17]. Although the accumulation of carbonylated proteins is a general marker of oxidative stress in damaged and aging tissue, the monitoring of specific protein modifications can provide additional insight into the nature and site of oxidative stress. The peroxiredoxin family of antioxidant thiol proteins, which includes the mitochondrial protein peroxiredoxin 3 (Prx 3), is responsible for breaking down endogenous hydroperoxides [18]. As such excess hydrogen peroxide can hyperoxidize the active site cysteine of Prxs, thereby inactivating the protein [19], [20], [21], [22].

Although many studies have documented early life nutritional influences on reproductive function [5], [23]–[26], mechanisms underlying these associations are unclear. To date, no data exist regarding maternal nutritional effects on adult offspring ovarian oxidant status. Therefore we set out to investigate and further characterise the effects of maternal undernutrition imposed at three different time periods: pregnancy (UNP), lactation (UNL) and pregnancy and lactation combined (UNPL), on offspring's ovarian morphology and gene expression levels. We also set out to determine whether nutritional-induced effects may be associated with disruptions in ovarian redox homeostasis. Given the importance of both the prenatal and postnatal environments contributing to disease development, we further investigated the effects of a mismatch between the prenatal and postnatal nutritional environments. Our study is the first to show that maternal UN significantly reduced primordial, secondary and antral follicle number in adult offspring in a manner that was dependent upon the timing of nutrient restriction. Specifically, a reduction in early stage follicle populations was observed in offspring born to mothers undernourished throughout both pregnancy and lactation, whereas antral follicles were reduced in offspring born to all mothers that were UN regardless of whether the period of UN was restricted to pregnancy or lactation or both. These reductions were associated with decreased mRNA levels of genes critical for follicle maturation and ovulation. We also demonstrate increased ovarian oxidative stress in offspring born to UN mothers regardless of the timing of the UN. However, only those mothers whose UN included the period of pregnancy produced offspring with reduced ovarian peroxidoxin 3 mRNA levels. This reduction may be suggestive of an inability to cope with increased oxidative stress conditions. Taken together, we propose that these data support the possibility that maternal UN, especially during the period of pregnancy, results in accelerated ovarian ageing in offspring. Intriguingly, feeding UN exposed offspring a post-weaning high fat (HF) diet, in general had little additional effect on ovarian physiology.

## Results

### Animal Phenotypic and Biochemical Data

#### Birth, weaning and adult body weights

*All females investigated in the current study were in the proestrous stage of cycle in order to minimise variation due to estrous stage.*

Maternal undernutrition resulted in a significant reduction in birth weight of UNP and UNPL offspring compared to Control and birth weight of UNL offspring was similar to that of Controls (Table 2). Maternal undernutrition resulted in a significant reduction in weaning weights of chow-fed and HF- fed offspring of all undernourished groups (p<0.001) (Table 2). Maternal undernutrition also resulted in significant reductions in adult offspring body weight (p = 0.006), with a further significant effect of a postnatal diet (p<0.001). There were no significant interaction effects between maternal and postnatal diets (p = 0.419). *Post-hoc* analysis demonstrated a statistically significant decrease in chow-fed and HF- fed offspring of mothers undernourished throughout pregnancy and lactation (UNPL, p<0.001; UNPL-HF, p<0.001) compared to chow-fed and HF- fed Controls. Adult offspring ovarian weights were similar between groups (Table 2).

**Table 1:** Primer sequences and amplicon sizes for the genes of interest.

	Primer Information			
Rat Gene	Forward Primer	Reverse Primer	Amplicon Length (bp)	GenBank Accession Number
Beta-actin	CACCAACTGGGACGATATGGA	CAGCCTGGATGGCTACGTACAT	188	NM_031144
Ob-Rb	GCTGCGTCATCCTTTCCT	TGGTTTTCCAACTCCTTCC	212	AF287268
ER-beta	TGCCAATCATCGCTCCTCTA	TCCTTCACATGACCAAACGC	228	AB190770.1
Pgr	CGGGAATTGATCAAGGCAAT	CCGGGATTGGATGAACGTAT	150	L16922.1
FSHR	TTGCTCCTGGTCTCCTTGCT	ACCTCAGTTCAATGGCGTTCC	151	NM199237
GDF-9	CCAAAGAGGGGGTTCCTAAA	CACAAGGTCACACACACAGG	245	NM021672
3beta-HSD	ACAGAAAGGGGGCAAGGATG	TGGACAGGGGATTAGGGAAGA	171	M38178
CYP17A1	ATCTTTGGGGCGGGCATAG	GTTAGCCTTGTGGGGGATGAG	234	NM012753
Prx3	GGCAAAACCTCACCATGCTT	GCTTCAGGGCAGGCTAAGAA	207	NM_022540.1

Abbreviations: Ob-Rb, Leptin receptor; Pgr, Progesterone receptor; FSHR, Follicle-stimulating hormone receptor; GDF9, Growth differentiation factor-9; Prx3, Peroxiredoxin 3; bp, base pairs.

**Table 2:** Phenotypic Data.

Groups	Birth Weights (g)	Weaning Weights (g)	Adult Body Weight (g)	Adult Ovary Weight (g)	Raw AG distance (mm)	AG % BW	AG % NA
Control	6.1±0.09	59.6±0.7	301.0±4.9	0.103±0.005	11.5±0.6	3.8±0.1	5.2±0.3
UNP	4.8±0.06 *	54.8±1.2 *	292.4±7.1	0.096±0.005	11.1±0.7	3.8±0.3	5.1±0.3
UNPL	4.8±0.08 *	32.7±1.3 *	263.3±6.6 *	0.097±0.006	11.3±0.3	4.3±0.2	5.2±0.1
UNL	6.0±0.08 *	39.7±1.2 *	292.6±15.6	0.100±0.006	11.0±0.3	3.82±0.2	4.9±0.2
Cont-HF	X	58.8±1.4	340.3±7.4	0.102±0.005	10.6±0.3	3.1±0.1	4.7±0.1
UNP-HF	X	54.0±1.8 #	358.5±13.4	0.105±0.005	12.4±0.6	3.5±0.2	5.4±0.2
UNPL-HF	X	35.7±1.5 #	311.6±13.2 #	0.094±0.006	12.6±0.5	4.1±0.2 #	5.6±0.2
UNL-HF	X	39.5±1.2 #	322.8±16.9	0.107±0.006	12.0±0.4	3.7±0.1 #	5.3±0.2

Data are presented as mean ± SEM. *Post-hoc* analyses:

*p<0.001 compared to Controls;

# p<0.05 compared to Cont-HF.

Abbreviations: AG, anogenital; BW, body weight; NA, nose to anus; NT, nose to tail.

#### Anogenital distance

There was no maternal diet effect (p = 0.265), postnatal diet effect (p = 0.066) and no significant interaction (p = 0.611) between the two factors on unadjusted offspring anogenital distance (AG) or on AG distance adjusted for body length (Table 2). However, there was a significant effect of maternal (p<0.001) and postnatal (p<0.001) diets on anogenital distance expressed as a percentage of body weight (AG % BW; Table 2). *Post-hoc* analysis demonstrated a statistically significant increase in AG % BW in HF fed offspring of UNP and UNPL mothers (p<0.001 for both).

#### Plasma Follicle stimulating hormone and inhibin B levels

Adult offspring proestrus concentrations of plasma FSH and inhibin B were not significantly different among treatment groups (data not shown).

### Maternal undernutrition modified offspring ovarian follicle number

#### Primordial and primary follicle number

**Figure 1:**
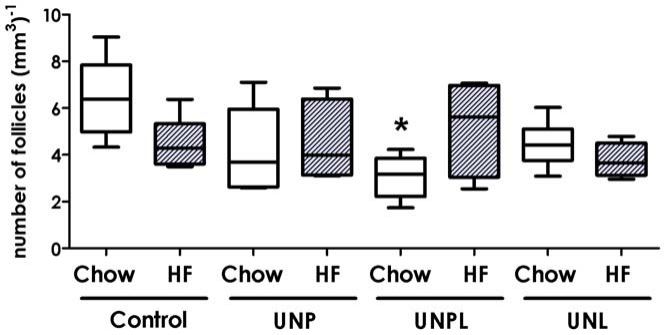
Effect of Maternal Undernutrition on Offspring Ovarian Primordial Follicle Number. Maternal UN occurring throughout pregnancy and lactation (UNPL) resulted in a significant reduction in offspring primordial follicle number. Data are presented as follicle number per mm3 of ovarian tissue; median ± upper and lower quartiles. Open bars correspond to a postnatal chow diet; closed bars correspond to a postnatal HF diet. Two-Way ANOVA Main Effects: maternal diet p = 0.035; postnatal diet  = 0.610; interaction p = 0.072. Holm-Sidak *Post-hoc* analyses: * p<0.001 compared to chow-fed Controls.

Primordial follicle number was significantly decreased in ovaries of offspring born to UN mothers (p = 0.035), with no effect of postnatal diet (p = 0.610) and no significant interactions between maternal and postnatal diet (p = 0.072) (Figure 1). *Post-hoc* analysis demonstrated a statistically significant decrease in primordial follicle number in chow-fed UNPL offspring compared to chow-fed controls (p<0.001, Figure 1). Post-weaning HF nutrition had no effect on primordial follicle number. There was no effect of maternal diet (p = 0.307), postnatal diet (p = 0.222) and no statistically significant interactions between maternal and postnatal diet (p = 0.336) on primary follicle number (data not shown).

#### Secondary follicle number

**Figure 2:**
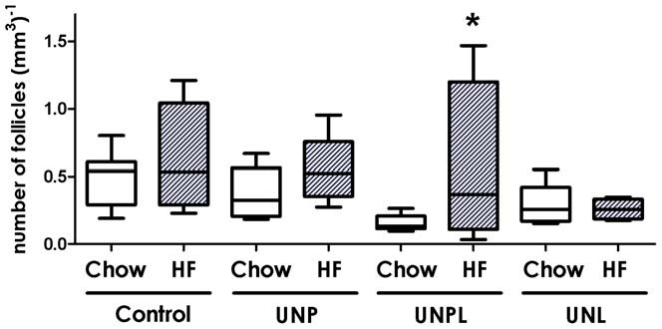
Effect of Maternal Undernutrition on Offspring Ovarian Secondary Follicle Number. Maternal UN occurring throughout pregnancy and lactation (UNPL) resulted in a significant reduction in offspring secondary follicle number. Data are presented as follicle number per mm3 of ovarian tissue; median ± upper and lower quartiles. Open bars correspond to a postnatal chow diet; closed bars correspond to a postnatal HF diet. Two-Way ANOVA Main Effects: maternal diet p = 0.004; postnatal diet  = 0.074; interaction p = 0.394. Holm-Sidak *Post-hoc* analyses: * p<0.001 compared to chow-fed Controls.

Secondary follicle number was significantly decreased in ovaries of offspring born to undernourished mothers (p = 0.004) (Figure 2), with no effect of postnatal diet (p = 0.074) and no significant interactions between maternal and postnatal diet (p = 0.394) (Figure 2). *Post-hoc* analysis demonstrated a statistically significant decrease in secondary follicle number in chow-fed UNPL offspring compared to chow-fed controls (UNPL; p<0.001) (Figure 2).

#### Antral follicle number

**Figure 3:**
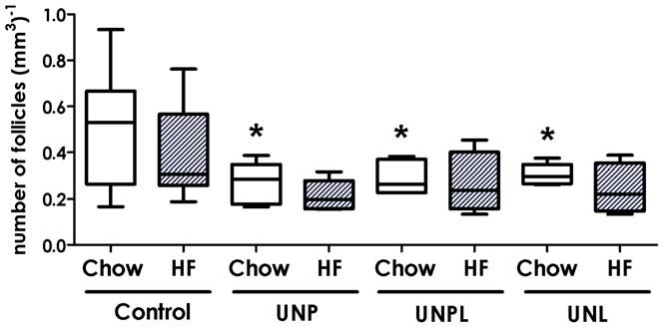
Effect of Maternal Undernutrition on Offspring Ovarian Antral Follicle Number. Maternal UN resulted in a significant reduction in offspring antral follicle number, regardless of the timing of nutrient restriction. Data are presented as follicle number per mm3; median ± upper and lower quartiles. Open bars correspond to a postnatal chow diet; closed bars correspond to a postnatal HF diet. Two-Way ANOVA Main Effects: maternal diet p = 0.005; postnatal diet  = 0.290; interaction p = 0.858. Holm-Sidak *Post-hoc* analyses: * p<0.005 compared to chow-fed Controls.

Antral follicle number was significantly decreased in ovaries of UN offspring (p = 0.005), with no effect of a postnatal diet (p = 0.290) and no interaction between maternal and postnatal diet (p = 0.858) (Figure 3). *Post-hoc* analysis demonstrated a statistically significant decrease in antral follicle number in chow-fed UNP offspring (p = 0.003), UNPL offspring (p = 0.008), and UNL offspring (p = 0.011) compared to chow-fed controls (Figure 3).

**Figure 4:**
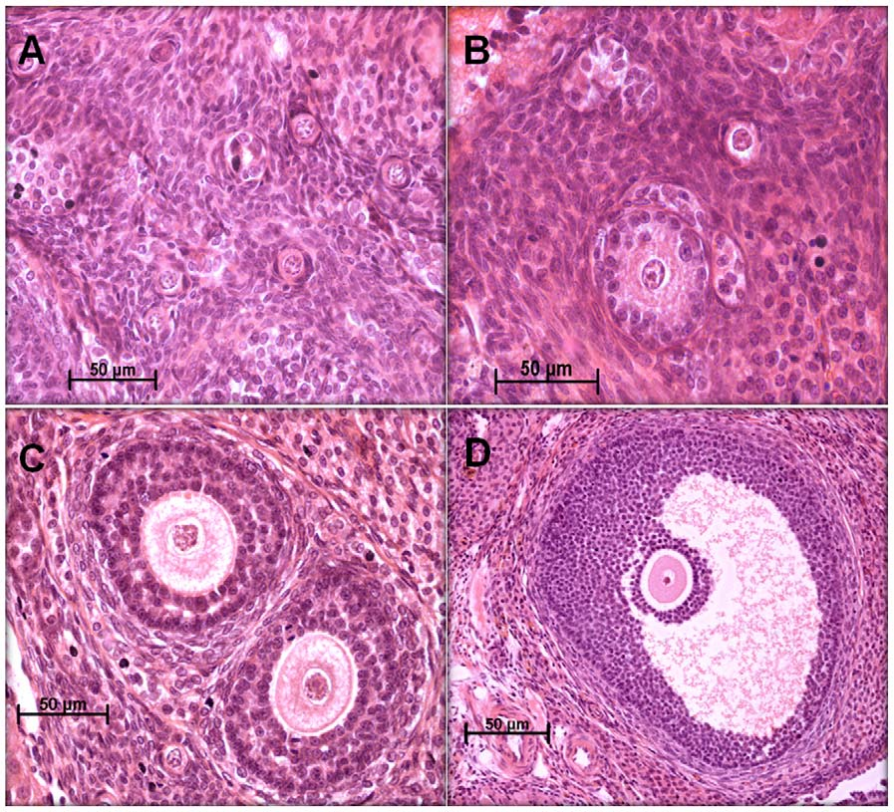
Morphological classification of rat ovarian follicles. Representative photographs of haematoxylin and eosin-stained ovarian tissue sections (8 µm) demonstrating representative classes of follicle populations. **A**) primordial, **B**) primary, **C**) secondary and **D**) antral follicles.

Morphological classification of follicles is illustrated in Figure 4.

### Maternal undernutrition alters offspring ovarian gene expression levels

#### Factors Involved in Folliculogenesis: Ovarian GDF-9, BMP-15, FSH-R, ER-β mRNA levels

**Figure 5:**
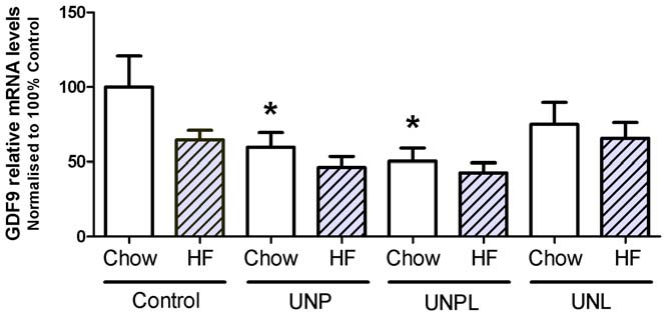
Effect of Maternal Undernutrition on Offspring Ovarian GDF-9 mRNA expression levels. Maternal UN occurring during pregnancy (UNP) and throughout pregnancy and lactation (UNPL) resulted in a significant reduction in ovarian GDF9 mRNA levels in chow-fed offspring. Data were normalised to beta-actin and relative mRNA data were normalised to 100% chow-fed Control. Data are presented as means ± S.E.M. Two-Way ANOVA Main Effects: maternal diet p = 0.004; postnatal diet  = 0.036; interaction p = 0.503. Holm-Sidak Post-hoc analyses: * p<0.05 compared to chow-fed Controls. # p = 0.016 compared to HF-fed Controls.

Ovarian growth differentiation factor 9 (GDF-9) mRNA levels were significantly decreased in ovaries from offspring born to UN mothers (p = 0.004), with a significant effect of postnatal diet (p = 0.036) (Figure 5). There were no significant interactions between maternal and postnatal diet (p = 0.503) (Figure 5). Post-hoc analysis demonstrated a statistically significant decrease in ovarian GDF-9 mRNA levels in chow-fed UNPL and UNP offspring (p = 0.002; p = 0.011) compared to chow-fed Controls (Figure 5). The consumption of a post-weaning HF diet resulted in a significant decrease in ovarian GDF9 mRNA levels in Control offspring (p = 0.016).

Ovarian bone morphogenetic protein-15 (BMP-15) mRNA levels were similar between Control and UN offspring, with no significant effect of maternal diet (p = 0.474), postnatal diet (p = 0.222) and no interaction between maternal and postnatal diets (p = 0.141) (Table 3).

**Table 3:** Relative mRNA levels of Genes of Interest Regulating Ovarian Folliculogenesis and Steroidogenesis.

	Genes of Interest				
Groups	BMP-15	FSHR	3β-HSD	CYP17A1	PR
Control	100±6.5	100±14.2	100±6.8	100±25.2	100±15.5
UNP	103.8±18.2	78.8±7.5	124.4±15.3	97.03±21.03	87.0±17.3
UNPL	78.2±8.7	101.4±11.5	95.0±8.7	51.1±9.2	85.0±10.2
UNL	89.3±14.7	190.2±20.5 *	82.8±11.4	142.6±31.3	182.3±29.2
Cont-HF	83.8±10.8	126.3±16.3	116.5±9.3	67.5±19.4	81.4±12.6
UNP-HF	60.3±9.0	65.3±5.6 #	102.4±8.2	71.2±20.9	54.2±9.0
UNPL-HF	77.2±8.5	79.1±23.8	113.9±28.7	75.9±33.0	85.0±16.8
UNL-HF	103.9±17.7	187.6±30.1 #	193.6±41.3 +	172.8±42.2	199.0±37.6 #
***Main Effects***					
*Maternal Diet*	*P = 0.474*	*P<0.001*	*P = 0.480*	*P = 0.032*	*P<0.001*
*Postnatal Diet*	*P = 0.222*	*P = 0.805*	*P = 0.013*	*P = 0.735*	*P = 0.262*
*Interaction*	*P = 0.141*	*P = 0.485*	*P = 0.004*	*P = 0.471*	*P = 0.370*

Data are mean ± SEM, relative to controls. *Post-hoc* analyses:

*p<0.001 compared to Controls;

# p<0.05 compared to Cont-HF;

+ p<0.001 UNL chow vs. UNL-HF. BMP15, bone-morphogenetic protein 15; FSHR, follicle-stimulating hormone receptor; 3β-HSD, 3beta dehydrogenase; CYP17A1, cytochrome P450 17A1; PR, progesterone receptor. Note: Multiple comparisons within UN groups are detailed in the text.

Maternal UN significantly altered ovarian follicle stimulating hormone receptor (FSHR) mRNA levels (p<0.001; Table 3). No significant effect of postnatal diet (p = 0.805) and no significant interactions between maternal and postnatal diets were observed (p = 0.485) (Table 3). *Post-hoc* analysis demonstrated a statistically significant increase in ovarian FSHR mRNA levels in chow-fed and HF-fed UNL offspring (p<0.001) compared to Control offspring. In contrast, a significant decrease in ovarian FSHR mRNA levels was observed in UNP-HF offspring (p = 0.006) compared to HF-fed controls (Table 3).

**Figure 6:**
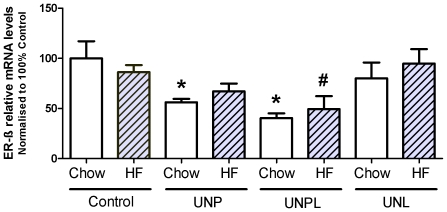
Effect of Maternal Undernutrition on Offspring Ovarian ER-β mRNA expression levels. Maternal undernutrition resulted in a significant reduction in offspring ovarian ER-β mRNA levels. Data were normalised to beta-actin and relative mRNA data were normalised to 100% chow-fed Controls. Data are presented as means ± S.E.M. Two-Way ANOVA Main Effects: maternal diet p<0.001; postnatal diet  = 0.585; interaction p = 0.424. Holm-Sidak *Post-hoc* analyses: * p<0.005 compared to chow-fed Controls. # p = 0.008 compared to HF-fed Controls.

There was a significant effect of maternal diet on ovarian estrogen receptor-beta (ER-β) mRNA levels (p<0.001), with no effect of a postnatal diet (p = 0.479) and no significant interactions between maternal and postnatal diet (p = 0.478) (Figure 6). *Post-hoc* analysis demonstrated a statistically significant decrease in ovarian ER-β mRNA levels in chow-fed UNP offspring (p = 0.005) and in UNPL offspring (p<0.001) compared to chow-fed controls (Figure 6). A significant decrease in ovarian ER-β mRNA levels was also observed in UNPL-HF-fed offspring (p = 0.008) compared to HF-fed Controls (Figure 6).

#### Factors Involved in Ovarian Steroidogenesis: 3β-HSD & CYP17A1 mRNA levels

Ovarian 3-beta hydroxysteroid dehydrogenase (3β-HSD) mRNA levels were not altered by maternal diet (p = 0.480), but a significant postnatal diet (p = 0.013) and maternal-postnatal diet interaction (p = 0.004) was observed (Table 3). *Post-hoc* analysis demonstrated a significant increase in ovarian 3β-HSD mRNA levels in HF-fed UNL offspring (UNL, chow vs. HF; p<0.001) (Table 3) compared to chow-fed UNL offspring (Table 3).

There was a significant effect of maternal diet (p = 0.032) on ovarian mRNA levels of P450 cytochrome A1 (CYP17A1), the steroid enzyme required to produce androstenedione which is an androgen and estradiol precursor. This difference however was restricted to UNPL versus UNL offspring (p = 0.005, Table 3). There was no significant effect of postnatal diet (p = 0.735) and no significant interaction between maternal and postnatal diets (p = 0.471) (Table 3).

#### Factors involved in Ovulation: Ovarian PR and Ob-Rb mRNA levels

Maternal UN altered ovarian PR mRNA levels in offspring (p<0.001), although differences were restricted to UNL-HF offspring versus Cont-HF offspring (p = 0.002). There was no significant effect of postnatal diet (p = 0.262) and no significant interaction effects between maternal and postnatal diets (p = 0.370) (Table 3).

**Figure 7:**
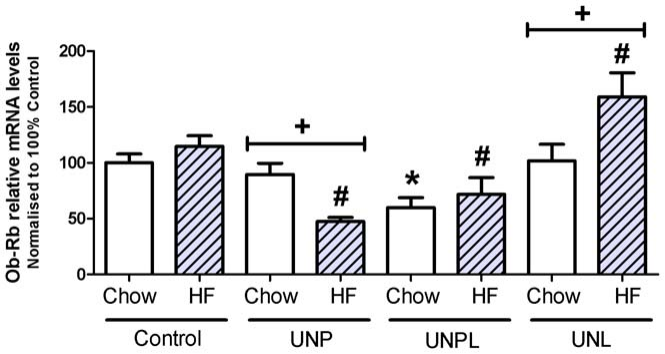
Effect of Maternal Undernutrition on Offspring Ovarian Ob-Rb mRNA expression levels. Maternal UN resulted in significant alterations in offspring ovarian Ob-Rb mRNA levels. Data were normalised to beta-actin and relative mRNA data were then normalised to 100% Control. Data are presented as means ± S.E.M.; n = 4–9 per group. Two-Way ANOVA Main Effects: maternal diet p<0.001; postnatal diet  = 0.165; interaction p = 0.001. Holm-Sidak *Post-hoc* analyses: * p<0.05 compared to chow-fed Controls; # p<0.05 compared to Cont-HF. + p<0.05, chow vs. HF.

Maternal UN significantly altered ovarian Ob-Rb mRNA levels in offspring (p<0.001), with no significant postnatal diet effect (p = 0.165). A significant interaction between maternal and postnatal diets (p = 0.001) was observed (Figure 7). *Post-hoc* analysis demonstrated a significant decrease in ovarian Ob-Rb mRNA levels in UNPL and UNPL-HF (p = 0.006) compared to chow-fed and HF-fed Controls respectively (Figure 7).

The consumption of a post-weaning HF-diet resulted in a significant decrease in ovarian Ob-Rb mRNA levels in UNP offspring (p<0.001) but an increase in mRNA levels was observed in UNL offspring (p = 0.004; Figure 7). Post-weaning HF nutrition did not alter Ob-Rb levels in any other groups.

### Maternal undernutrition modified markers of oxidative stress

#### Ovarian protein carbonyl content

**Figure 8:**
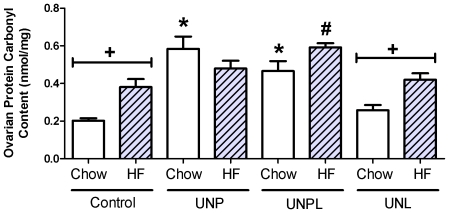
Effect of Maternal Undernutrition on Offspring Ovarian Protein Carbonyl Content. Maternal UN resulted in a significant increase in ovarian protein carbonyl content in offspring, irrespective of the timing of nutrient restriction. Ovarian protein carbonyl content (nmol/mg of ovarian tissue); Data are presented as means ± S.E.M, Two-Way ANOVA Main Effects: maternal diet p<0.001; postnatal diet  = 0.008; interaction p = 0.001). Holm-Sidak *Post-hoc* analyses: * p<0.001 compared to chow-fed Controls. # p = 0.003 compared to Cont-HF. + p<0.05, chow vs. HF.

**Figure 9:**
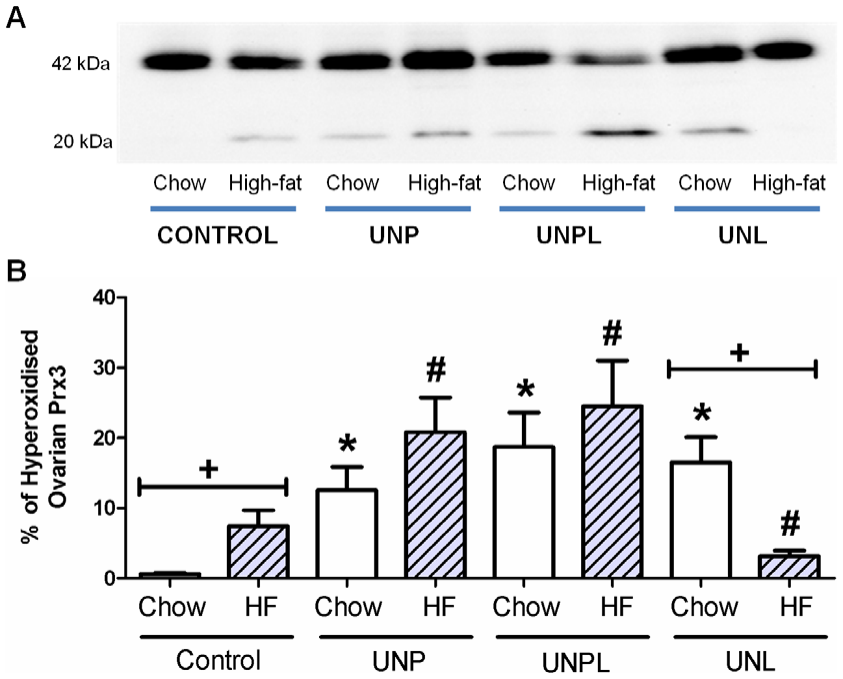
Effect of Maternal Undernutrition on the Presence of Ovarian Hyperoxidised Prx 3. Maternal UN resulted in the increased presence of hyperoxidised ovarian Prx 3 in chow-fed UNP and UNPL, but not UNL, offspring. **A**) Representative non-reducing Western blot of Prx 3; Prx 3 is visualised as a 42 kDa band, hyperoxidised Prx 3 is visualised as a low molecular weight band (20 kDa); **B**) Data are presented as % hyperoxidised ovarian Prx 3 (as a ratio of band densities) and graphed as means ± S.E.M.; Two-Way ANOVA Main Effects: maternal diet p<0.001; postnatal diet  = 0.036; interaction p<0.001. Holm-Sidak *Post-hoc* analyses: * p<0.001 compared to chow-fed Controls. # p<0.001 compared to Cont-HF. + p<0.05, chow vs. HF.

To assess global oxidative stress, protein carbonyls in ovarian tissue samples were measured by ELISA. Maternal UN (p<0.001) and postnatal diet (p = 0.008) significantly altered protein carbonyl content and a statistically significant interaction between maternal and postnatal diets (p = 0.001) was observed (Figure 8). *Post-hoc* analysis demonstrated a three-fold increase in ovarian protein carbonyl levels in chow-fed UNP (p<0.001), and UNPL offspring (UNPL; p<0.001), compared to chow-fed Controls (Figure 8). This effect was absent in UNL offspring (Figure 9). The consumption of a post-weaning HF diet resulted in a significant increase in protein carbonyl levels in Control and UNL offspring, but had no further effect on UNP and UNPL levels (Figure 8).

#### Presence of hyperoxidised peroxiredoxin 3 (Prx 3)

Mitochondrial oxidative stress was assessed by the presence of hyperoxidised Prx 3 in ovarian tissue. Reduced Prx 3 is oxidized to an intermolecular dimer (42 kDa) following cell lysis, while the hyperoxidized Prx 3 remains as a monomer (20 kDa), as visualised by Western blotting [27]. There was a marked absence of hyperoxidised Prx 3 in chow fed Control offspring (Figure 9A). Maternal UN (p<0.001) and postnatal diet (p = 0.036) resulted in a significant increase in the presence of ovarian hyperoxidised Prx 3 protein levels (Figure 9B). A statistically significant interaction between maternal and postnatal diet (p<0.001) was also observed. *Post-hoc* analysis demonstrated a significant increase in ovarian hyperoxidised Prx 3 in UNP, UNPL and UNL offspring compared to Controls (Figure 9B). A post-weaning HF diet significantly increased ovarian hyperoxidised Prx 3 in Control offspring (Cont-HF; p<0.001) and although levels were higher in UNP-HF and UNPL-HF offspring compared to Cont-HF offspring, these were not different from their respective chow fed counterparts. In contrast, hyperoxidised Prx 3 levels were significantly decreased in UNL-HF offspring (p<0.001) (Figure 9B) compared to HF-fed Control.

#### Ovarian Peroxiredoxin 3 mRNA levels

**Figure 10:**
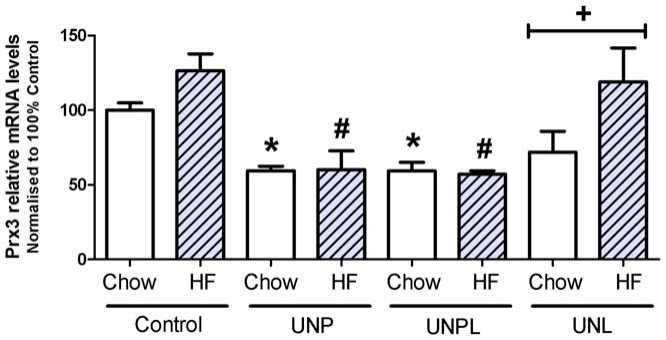
Effect of Maternal Undernutrition on Offspring Ovarian Prx 3 mRNA expression. Maternal UN resulted in a significant reduction in ovarian Prx 3 mRNA levels in chow-fed UNP and UNPL offspring. Data were normalised to beta-actin and relative mRNA data were normalised to 100% Controls. Data are presented as means ± S.E.M. Two-Way ANOVA Main Effects: maternal diet p<0.001; postnatal diet  = 0.023; interaction p = 0.111. Holm-Sidak *Post-hoc* analyses: * p<0.05 compared to chow-fed Controls. # p<0.05 compared to Cont-HF. + p<0.05, chow vs. HF.

Maternal UN (p<0.001) and postnatal diet (p = 0.044) significantly altered ovarian Prx 3 mRNA levels, although no significant interaction between maternal and postnatal diet was observed (p = 0.202; Figure 10). *Post-hoc* analysis demonstrated a significant decrease in Prx 3 mRNA levels in chow-fed UNP and UNPL offspring (p = 0.010 and p = 0.004) compared to chow-fed Controls (Figure 10) and although levels were lower in UNL offspring these differences did not reach statistical significance (p = 0.242). Although a post-weaning HF diet had no further effect on Prx 3 mRNA levels in UNP and UNPL offspring (Figure 10), post-weaning HF nutrition significantly increased ovarian Prx 3 mRNA expression in UNL offspring (p = 0.010) (Figure 10) and tended to increase levels in control offspring although this difference did not reach statistical significance (p = 0.066).

## Discussion

These data demonstrate that maternal undernutrition (UN), imposed at defined critical windows of development and resulted in significant reductions in primordial, secondary and antral follicle number in a manner that was dependent on the timing of nutrient restriction (see Table 4, summary). These changes may be underpinned by observed alterations in the levels of key genes involved in folliculogenesis and may be the result of increased ovarian oxidative stress and possible impairment of a major mitochondrial antioxidant defense network. These offspring are born growth restricted and demonstrated catch-up growth in weight by adulthood, which was exacerbated by postweaning high fat nutrition. Thus, as shown by us and others, in the presence of excess neonatal nutrition, growth-restricted pups born to undernourished mothers demonstrate catch-up growth, such that their body weights match or exceed those of controls early in postnatal life [28].

**Table 4:** Summary of results.

		UNP	UNPL	UNL
***Follicle Number***	Primordial		**↓**	
	Secondary		**↓**	
	Antral	**↓**	**↓**	**↓**
***Gene Expression***	GDF9	**↓**	**↓**	
	ER-β	**↓**	**↓**	
	Ob-Rb		**↓**	
	Prx3	**↓**	**↓**	
***Oxidative Stress Markers***	Protein Carbonyls	**↑**	**↑**	
	Hyperoxidised Prx 3	**↑**	**↑**	**↑**

Summary of results of follicle number counts, mRNA analyses and oxidative stress measures in ovaries of offspring born to mothers undernourished during pregnancy (UNP), during pregnancy and lactation (UNPL) and during lactation alone (UNL). Arrows up indicate a significant (p<0.05) increase compared to Controls; arrows down indicate a significant (p<0.05) decrease compared to Controls. Results in table represent offspring that were fed a post-weaning control diet. For results in offspring fed a post-weaning high fat diet please see text.

### Maternal undernutrition

The negative impact of maternal UN on reproductive function has been documented by our group [5] and others [23], [24], [29], [30] but the effects of early life nutritional adversity on ovarian follicular development remains unclear. We have previously reported that pre- and postnatal nutritional histories together influence both ovarian function and the tempo of reproductive maturation in female offspring, where diminished maternal calorie intake resulted in early pubertal onset and reduced adult plasma progesterone levels [5]. We speculated that these effects may be interpreted as a life history trade-off for earlier reproductive maturation but accompanied by a faster decline in ovarian function with aging. We now demonstrate that offspring born to UN mothers have characteristics of accelerated ovarian aging and reduced ovarian reserve. This is consistent with data that suggest that aging *per se* may be accelerated in offspring of poorly nourished dams. In this regard, Ozanne et al. have reported that prenatal UN, in contrast to postnatal UN, leads to reduced longevity in mice [31], [32].

In the rat ovary, the first growing primordial follicles are found in the highly innervated, richly vascularised corticomedullary junction, and primordial follicle activation and subsequent follicular growth depends on nutrients, hormones and/or growth factors [33]. UN during the period before weaning has been shown to impact folliculogenesis [34], and therefore it is likely that our observed reduction in early stage follicle number was a result of the direct action of UN on the ovary. GDF-9, a key oocyte-derived member of the transforming growth factor β (TGF-β) family, plays an essential role in early folliculogenesis [35] and our findings may suggest therefore that reduced ovarian GDF-9 mRNA levels in UNPL and UNP offspring contributed to a disruption in early follicle growth. This is in agreement with previous studies showing that GDF-9 is required for primordial to primary transition [36] and GDF-9 enhances the progression of early to late-stage primary follicles in the rat [37]. GDF-9 has also been shown to suppress granulosa cell apoptosis and follicular atresia during the preantral-early antral transition [38]. Therefore our observed reductions in GDF-9 levels may inhibit follicular survival and growth during the progression to early antral stage, potentially contributing to the reduction in antral follicle number. These findings are consistent with previous reports where lactational UN resulted in a reduction in primordial and large antral follicles [24].

Diminished intraovarian action of estradiol via decreased ovarian ER-β levels in UNP and UNPL chow-fed offspring may also negatively impact antral follicle number. Estradiol has been shown to increase follicular expression of both FSH and LH receptors in rat granulosa cells [39] and increase central leptin sensitivity and leptin receptor expression [40]. In this regard, reduced ovarian ER-β mRNA levels may contribute to altered Ob-Rb mRNA levels and downstream activity. As ovarian leptin is important for ovulation, oocyte maturation and steroidogenesis [9], [41]–[44], the reduction in Ob-Rb mRNA levels combined with decreased ER-β mRNA levels could together contribute to the observed decline in antral follicle number in UNPL offspring. FSH and its receptor (FSHR) play an important role in follicle progression from preantral to preovulatory stage particularly in rodents [45]. Interestingly, ovarian FSHR levels were unchanged in chow-fed UNP and UNPL offspring but were increased in chow-fed UNL offspring. These findings are unexpected and is not consistent with previous studies [23] as a rise in ovarian FSHR mRNA levels would be anticipated to be concomitant with an increase in antral follicle number. These changes were not accompanied by changes in circulating FSH levels in these offspring. Since circulating 17β-estradiol levels were not measured in this study, we can only speculate that possibly the observed increase in FSHR levels in UNL offspring may be a result of other hormonal mechanisms, including altered 17β-estradiol levels.

We recognize that the use of whole ovarian tissue to measure mRNA levels of key genes may have masked any discernable changes specific to follicle populations, had they been processed and examined separately. However, given that we have demonstrated robust changes in key follicular regulatory factors, we suggest that our methods have not limited our results. Nevertheless, due to previously reported differential expression of key factors in follicle subtypes [37], [39], [44], it is difficult to discern whether our observed changes in follicle number after maternal nutrient restriction were due to changes in receptor/growth factor function or; whether our observed changes in mRNA levels were due to differences in follicle number. These data do however provide strong evidence for examining follicle subtypes individually and this will be considered in future studies.

Reactive oxygen species play a role in the modulation of an entire spectrum of reproductive functions including oocyte maturation, ovarian steroidogenesis, corpus luteal function and luteolysis [46]. Oxidative stress and disruption of mitochondrial function is a trigger of apoptosis [47], contributing to the exhaustion of oocyte reserve, either directly through germ cell death or indirectly through follicular atresia [48], [49]. Early life nutritional adversity has been associated with increased oxidative stress [15], [50], [51], [52]. The ovary is a metabolically active organ and reactive oxygen species will be generated during normal physiological functioning. Superoxide dismutase levels have been positively correlated to ovarian steroidogenesis [53], and pre-ovulatory antral follicles demonstrate lipid peroxidation [54] and regulation of follicular hydroperoxides may be mediated by glutathione peroxidase [55]. As protein carbonyl levels are common markers of protein oxidation our data suggest that maternal UN particularly during the critical time during pregnancy results in a significant increase in global ovarian oxidative stress. We did not observe an accumulation of protein carbonyls in ovaries of UNL offspring, suggesting that undernourishment during pregnancy is indeed the driver for increased global oxidation. These data are consistent with the observation that both UNP and UNPL offspring demonstrated significant decreases in ovarian follicle number and in specific gene expression levels.

We also assessed changes in the redox status of mitochondrial Prx 3. A significant reduction in mitochondrial Prx 3 mRNA levels was observed in the ovaries of UNP and UNPL, which may translate to a decreased capacity of this antioxidant system within the ovary. This observation was consistent with an increased presence of ovarian hyperoxidised Prx 3, which is catalytically inactive and only slowly recycled to the active form [18]. Previous studies have reported that depletion of Prx 3 sensitises cells to oxidative stress and apoptosis, and cells with depleted Prx 3 exhibit increased protein carbonylation [56]. Prx 3 hyperoxidation has been reported in the liver of aged rats [21] and the accumulation of damage exerted by increased levels of reactive oxygen species has also been implicated in ovarian aging [57], [58]. Although our study does not measure causality, we speculate that increased oxidative stress directly contributed to the altered primordial, secondary and antral follicle number observed in the ovaries of UN offspring. Consistent with this, estradiol has been reported to act as a survival factor against follicle atresia and studies have reported estrogen-induced inhibition of granulosa cell apoptosis in rat antral follicles [59]. It is possible that, increased ovarian oxidative stress and decreased ERβ mRNA levels combine to promote follicular apoptosis and atresia in UNP and UNPL offspring. Together, these data present the possibility that the offspring of UN mothers may have exhibited accelerated ovarian aging, thereby contributing to the decline in follicle reserve.

### Maternal undernutrition combined with post-weaning high fat nutrition

We have demonstrated that maternal nutrient restriction resulted in a decrease in ovarian follicle number, changes in gene expression of key ovarian regulators, and enhanced ovarian oxidative stress in the offspring. Interestingly there were only two instances where the prenatal and postnatal nutritional environments interacted to affect our measurable outcomes: mRNA levels of 3β-HSD and Ob-Rb. Both of these factors have functional roles in steroidogenesis [9], [60], [61] and we have recently shown that in the adrenal gland, a postnatal HF diet was needed to elicit effects on key enzymes in the steroidogenic pathway [61]. A postnatal HF diet significantly altered ovarian Ob-Rb as well as 3β-HSD mRNA levels and ovarian oxidative stress in a manner that was not only dependent upon maternal nutritional background, but also dependent upon on the critical period of UN exposure. UNP–HF offspring demonstrated a reduction in ovarian Ob-Rb mRNA levels. Conversely, UNL-HF offspring demonstrated a significant increase in ovarian Ob-Rb levels. It is tempting to speculate that UNL-HF offspring may have altered ovarian leptin signaling, as previous work has shown that HF-fed UN offspring had elevated levels of circulating plasma leptin compared to HF-fed Controls [5], [62] but further studies are required to investigate the leptin signalling pathway within these ovaries.

Oxidative stress has been previously implicated in HF diet-induced obesity [63]–[65]. Recently, maternal obesity has been shown to result in altered oocyte redox homeostasis and altered mitochondrial activity in zygotes [15]. In the current study we found that the imposition of a post-weaning HF diet increased oxidative stress conditions in Control and UNL groups only, where no additional affect was observed in UNP and UNPL offspring. It is possible that the levels of oxidative stress reached a threshold in UNP and UNPL chow-fed offspring and that post-weaning HF nutrition had no further effect. This also may be the case in our observed suppression of Prx 3 mRNA levels. However, it remains apparent that UN during pregnancy resulted in modifications in ovarian development and influenced ovarian responses to a HF challenge.

### Conclusions

We present novel data that maternal UN during pregnancy and/or lactation significantly altered ovarian follicular populations in adult offspring that was accompanied by changes in estrogen and leptin receptor levels. These alterations were dependent upon the timing of maternal UN. Our data suggest that these changes may be associated with increased ovarian oxidative stress and decreased ability to repair the resultant oxidative damage. These data contribute to the known association between oxidative stress and reproductive disorders, and suggest that early life nutrition may mediate this association.

## Methods

### Animal Model

**Figure 11:**
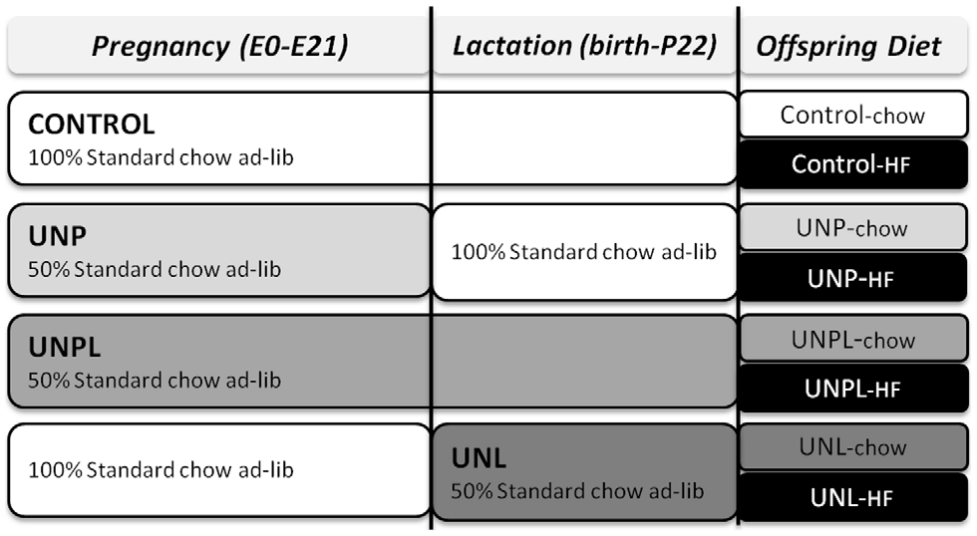
Experimental Design. Schematic representation of the experimental design. There are four levels of maternal nutrition and 2 levels of post-weaning diet resulting in a total of 8 experimental groups in a fully balanced 4×2 design.

In the present study, we used an established model of developmental programming via maternal global nutrient manipulation [5]. Wistar rats (postnatal day 120) were time mated using a rat estrous cycle monitor to assess the stage of estrous before introducing the male. After confirmation of mating, rats were housed individually in standard rat cages with free access to water. All rats were kept in the same room with a constant temperature maintained at 25°C and a 12-h light, 12-h dark cycle. Pregnant dams were randomised into one of 4 nutritional groups: 1) dams fed a standard control diet (protein 18%, fat 5%, digestible energy 3.4 kcal/gm, Teklad Global 18% Protein Diet, Diet 2018) *ad libitum* throughout pregnancy and lactation (**Control**), 2) undernourished dams fed 50% of Control intake throughout pregnancy and lactation (**UNPL group**), 3) undernourished dams fed 50% of Control intake throughout pregnancy only (**UNP group**) and 4) undernourished dams fed 50% of Control intake throughout lactation only (**UNL group**). After birth, pups were weighed and litter size adjusted to eight pups (4 male, 4 female) per litter to ensure standardised nutrition until weaning at day 22. At weaning, all offspring were weight matched within maternal dietary groups and placed on either the standard control rat chow or a HF diet (**Chow**, **HF**; D12451, 45% kcals as fat, Research Diets Inc., NJ, USA), resulting in a total of 8 experimental groups (Figure 11). At 150 days of postnatal age, all offspring were fasted overnight and killed by decapitation following pentobarbitone anaesthesia (60 mg/kg, s.c.). While under anaesthesia, the anogenital (AG) distance was measured in all offspring and vaginal smears were performed in females for determination of estrous stage at the time of tissue collection [5]. Both ovaries were collected; one was fixed in Bouin's Solution (Sigma Aldrich, cat HT101128 – 85.1% H_2_0, 9% formaldehyde, 5% acetic acid, 0.9% picric acid) and processed for later morphological analysis and the other snap-frozen in liquid nitrogen and stored at -80°C for subsequent RNA extraction and molecular analyses. All animal experiments were approved under guidelines of the Animal Ethics Committee at the University of Auckland (R402).

### Circulating levels of FSH and inhibin B

Circulating concentrations of FSH and inhibin B were measured using commercially available rat-ELISA kits (FSH, Biocode Hycel AER004, Immunodiagnostic Systems, UK; inhibin B, Beckman Coulter (Diagnostic Systems Laboratories) 10-84100) as per the manufacturer's specifications.

### Morphometric Analyses of Follicle Populations

Fixed ovaries were processed for microscopy and subsequently the entire ovary was sectioned at 8 µm. Every 5^th^ ovarian section was stained with haematoxylin and eosin for morphometric analysis. In order to prevent multiple counts of the same follicle, only those follicles with a visible oocyte nucleus were included. Since oocyte nuclei measured between 20–30 µm in diameter, counting every 5th section of the ovary ensured a distance of 40 µm between analysed sections, preventing multiple counts of the same ovarian follicle. Follicle classification based on characteristics proposed by Hirshfield & Midgley [66] was as follows: type 1: *primordial follicle*, one layer of flattened granulosa cells surrounding the oocyte; type 2: *primary follicle*, one to fewer than two complete layers of cuboidal granulosa cells; type 3: *secondary follicle*, an oocyte surrounded by greater than one layer of cuboidal granulosa cells, with no visible antrum; type 4: *antral follicle*, an oocyte surrounded by multiple layers of cuboidal granulosa cells and containing one or more antral spaces, cumulus oophorus and theca layer may also have been evident. Total volume of each section was calculated (area of the section x thickness of the section) and follicle counts for each animal were corrected for the total volume of ovarian tissue counted. All follicle counts were then expressed as number of follicles counted per mm^3^ of ovarian tissue counted.

### Molecular Analyses

#### RNA extraction and reverse transcription (RT)

Total RNA was extracted using commercially available kits (AllPrep DNA/RNA Mini kit; Qiagen, cat 80204). Genomic DNA contamination was removed from each sample via treatment with RNase-free DNase (Invitrogen Life Technologies; Auckland, NZ) according to the manufacturer's instructions. RNA quantity and purity were analysed using a NanoDrop spectrophotometer (ND-1000; BioLab Ltd) using NanoDrop software (version 3.1.2). All RNA samples were stored at −80°C until required.

5 µg of total RNA was used for first strand cDNA synthesis using the Moloney Murine Leukaemia Virus Reverse Transcriptase enzyme (M-MLV-RT) (Promega Corp, Wisconsin USA) and a standard thermocycler (GeneAmp® PCR System 9700, Applied Biosystems, California, USA). A master mix was prepared containing the following: 5 µL M-MLV 5× buffer (In Vitro Technologies, cat M531A), 0.5 µL M-MLV-RT (In Vitro Technologies, cat M170B) and 1.25 µL 10 µM deoxynucleosides triphosphates (dNTPs) (Global Science, cat R0181) under the following cycling conditions: an initial denaturation stage of 5 minutes at 96°C, followed by 30 cycles of 30 seconds each of 96°C (denaturation), 60°C (annealing stage) and 72°C (extension stage). cDNA was stored at −20°C for later use in qPCR assays.

#### Quantitative Polymerase Chain Reaction (qPCR) Assays

For the quantification of ovarian gene expression levels and of the endogenous reference beta actin, a quantitative PCR assay was performed using the ABI PRISM® 7900HT Sequence Detection System (Applied Biosystems; Auckland, New Zealand). All primers were designed using Primer 3 software (version 0.4.0, Whitehead Institute for Biomedical Research (Steve Rozen and Helen J. Skaletsky (2000) Primer3 on the WWW for general users and for biologist programmers. In: Krawetz S, Misener S (eds) Bioinformatics Methods and Protocols: Methods in Molecular Biology. Humana Press, Totowa, NJ, pp 365–386) (Table 1) and manufactured by Invitrogen (Invitrogen Life Technologies; Auckland, New Zealand). Optimal primer conditions were adjusted to the following cycling conditions: Length: 20 bp (range 17–23 bp), Tm: 63°C (range 60–65°C), and amplicon length: 100–300 bp. Dissociation analyses were performed to ensure specificity and samples producing a single peak in the dissociation curves were used. Amplified products were visualised on an agarose gel using the E-Gel® CloneWell 0.8% SYBR Safe gel (Invitrogen, cat G6618-08), run on the E-Gel® iBase™ Power System (Invitrogen, cat G6400) and sequenced by spectrophotometry (Allan Wilson Centre, Massey University). The resulting sequences were evaluated using NCBI BLAST to ensure specificity.

Quantification of gene expression levels were performed under the following conditions: an initial 2 min hold period at 50°C for normalisation (Stage 1), followed by enzyme activation at 95°C for 2 min (Stage 2); amplification of the gene product through 40 successive cycles of 95°C for 15 sec then 60°C for 1 min (Stage 3); followed by a dissociation stage of 15 sec at 95°C, 15 sec at 60°C and 2 min at 99°C (Stage 4). A standard curve was generated from the mean cycle threshold (Ct) of eight standards (1∶5 serial dilution) of a known concentration in triplicate, while amplification and dissociation curves were generated for all standards and samples (Applied Biosystems, California, USA). Each sample was run in triplicate.

### Measurement of oxidative stress

Frozen ovarian tissue was thawed and homogenized in 100 µl of phosphate buffered saline (PBS) containing 10 µM diethylene triamine pentaacetic acid (DTPA) and 20 µM of butylated hydroxytoluene (BHT). Protein concentration was measured using the Bradford dye-binding procedure and expressed as µg/µL. Fifty to sixty µL of the homogenate was set aside for protein carbonyl detection. The remainder was treated with Complete™ protease inhibitor (Roche Applied Science, Mannheim, Germany) and used for the detection of Prx 3 hyperoxidation using Western blotting.

#### Protein carbonyls

Protein carbonyl concentrations were determined by an enzyme-linked immunosorbent assay (ELISA) after derivatization with 2,4-dinitrophenylhydrazine using the low protein method suggested in the BioCell Protein Carbonyl Assay kit (BioCell, Papatoetoe, New Zealand).

#### Hyperoxidied Prx 3

Protein samples (10 µg) were separated on 12% SDS-polyacrylamide gel under non-reducing conditions and blotted onto Hybond PVDF membranes (GE Healthcare Life Sciences, Piscataway, NJ, USA). After transfer, the membranes were blocked in 5% skim milk TBS-T_20_ (150 mM NaCl, 50 mM Tris-HCl, pH 8.0, and 0.05% Tween 20) for 1 hour at room temperature and incubated overnight at 4°C with 1∶10,000 rabbit anti-Prx 3 polyclonal antibody (Ab Frontier, Seoul, Korea). Following washing, membranes were incubated with peroxidase-conjugated goat anti-rabbit secondary antibody and visualized using the ECL Plus Western Blotting Detection Reagents (GE Healthcare Life Sciences, Piscataway, NJ, USA) in combination with the Chemidoc XRS gel documentation system and Quantity One software (BioRad laboratories, Hercules, CA, USA) to quantify the density of the relevant bands.

### Statistical Analyses

All data were analysed by two-way factorial ANOVA, with maternal diet and offspring postnatal diet as factors. In all cases, data that were not normally distributed were log transformed to achieve data normality. Post-ANOVA comparisons among means were made using the Holm-Sidak method where appropriate. Follicle data are presented as box plots, where the box represents 25^th^ and 75^th^ percentiles (the lower and upper quartiles, respectively), and within which is shown the 50^th^ percentile (the median). The whiskers indicate the upper and lower values not classified as statistical outliers. In all cases, relative mRNA levels of the genes of interest were normalised to 100% of chow-fed offspring of Control mothers (Control). A p-value of <0.05 was considered statistically significant and all data are presented as means ± S.E.M. All statistical evaluation was performed using SigmaStat for Windows version 2.03 (Jandel Corp., San Ramon, CA, USA.).

## References

[1] Godfrey KM, Gluckman PD, Hanson MA (2010). Developmental Origins of Metabolic Disease: Life Course and Intergenerational Perspectives.. Trends Endocrinol Metab.

[2] Gluckman PD, Hanson MA, Morton SM, Pinal CS (2005). Life-Long Echoes–a Critical Analysis of the Developmental Origins of Adult Disease Model.. Biology of the Neonate.

[3] Gluckman PD, Hanson MA, Cooper C, Thornburg KL (2008). Effect of in Utero and Early-Life Conditions on Adult Health and Disease.. N Engl J Med.

[4] Sloboda DM, Hart R, Doherty DA, Pennell CE, Hickey M (2007). Age at Menarche: Influences of Prenatal and Postnatal Growth.. J Clin Endocrinol Metab.

[5] Sloboda DM, Howie GJ, Pleasants A, Gluckman PD, Vickers MH (2009). Pre- and Postnatal Nutritional Histories Influence Reproductive Maturation and Ovarian Function in the Rat.. PLoS One.

[6] Gardner DS, Ozanne SE, Sinclair KD (2009). Effect of the Early-Life Nutritional Environment on Fecundity and Fertility of Mammals.. Philos Trans R Soc Lond B Biol Sci.

[7] Bluher S, Mantzoros CS (2007). Leptin in Reproduction.. Curr Opin Endocrinol Diabetes Obes.

[8] Barkan D, Hurgin V, Dekel N, Amsterdam A, Rubinstein M (2005). Leptin Induces Ovulation in Gnrh-Deficient Mice.. FASEB J.

[9] Barkan D, Jia H, Dantes A, Vardimon L, Amsterdam A (1999). Leptin Modulates the Glucocorticoid-Induced Ovarian Steroidogenesis.. Endocrinology.

[10] Cheung CC, Thornton JE, Kuijper JL, Weigle DS, Clifton DK (1997). Leptin Is a Metabolic Gate for the Onset of Puberty in the Female Rat.. Endocrinology.

[11] Ahima RS, Dushay J, Flier SN, Prabakaran D, Flier JS (1997). Leptin Accelerates the Onset of Puberty in Normal Female Mice.. The Journal of Clinical Investigation.

[12] Kiess W, Blum WF, Aubert ML (1998). Leptin, Puberty and Reproductive Function: Lessons from Animal Studies and Observations in Humans.. Eur J Endocrinol.

[13] Theys N, Clippe A, Bouckenooghe T, Reusens B, Remacle C (2009). Early Low Protein Diet Aggravates Unbalance between Antioxidant Enzymes Leading to Islet Dysfunction.. PLoS One.

[14] Loui A, Raab A, Maier RF, Bratter P, Obladen M (2009). Trace Elements and Antioxidant Enzymes in Extremely Low Birthweight Infants.. J Trace Elem Med Biol.

[15] Igosheva N, Abramov AY, Poston L, Eckert JJ, Fleming TP (2010). Maternal Diet-Induced Obesity Alters Mitochondrial Activity and Redox Status in Mouse Oocytes and Zygotes.. PLoS One.

[16] Dalle-Donne I, Rossi R, Giustarini D, Milzani A, Colombo R (2003). Protein Carbonyl Groups as Biomarkers of Oxidative Stress.. Clin Chim Acta.

[17] Chakravarti B, Chakravarti DN (2007). Oxidative Modification of Proteins: Age-Related Changes.. Gerontology.

[18] Cox AG, Winterbourn CC, Hampton MB (2010). Mitochondrial Peroxiredoxin Involvement in Antioxidant Defence and Redox Signalling.. Biochem J.

[19] Rabilloud T, Heller M, Gasnier F, Luche S, Rey C (2002). Proteomics Analysis of Cellular Response to Oxidative Stress. Evidence for in Vivo Overoxidation of Peroxiredoxins at Their Active Site.. J Biol Chem.

[20] Woo HA, Chae HZ, Hwang SC, Yang KS, Kang SW (2003). Reversing the Inactivation of Peroxiredoxins Caused by Cysteine Sulfinic Acid Formation.. Science.

[21] Musicco C, Capelli V, Pesce V, Timperio AM, Calvani M (2009). Accumulation of Overoxidized Peroxiredoxin Iii in Aged Rat Liver Mitochondria.. Biochim Biophys Acta.

[22] Cox AG, Peskin AV, Paton LN, Winterbourn CC, Hampton MB (2009). Redox Potential and Peroxide Reactivity of Human Peroxiredoxin 3.. Biochemistry.

[23] da Silva Faria T, de Bittencourt Brasil F, Sampaio FJ, da Fonte Ramos C (2010). Maternal Malnutrition During Lactation Affects Folliculogenesis, Gonadotropins, and Leptin Receptors in Adult Rats.. Nutrition.

[24] da Silva Faria T, de Bittencourt Brasil F, Sampaio FJ, da Fonte Ramos C (2010). Effects of Maternal Undernutrition During Lactation on Estrogen and Androgen Receptor Expressions in Rat Ovary at Puberty.. Nutrition in press, corrected proof.

[25] Rae MT, Kyle CE, Miller DW, Hammond AJ, Brooks AN (2002). The Effects of Undernutrition, in Utero, on Reproductive Function in Adult Male and Female Sheep.. Anim Reprod Sci.

[26] Hilakivi-Clarke L, Clarke R, Lippman M (1999). The Influence of Maternal Diet on Breast Cancer Risk among Female Offspring.. Nutrition.

[27] Cox AG, Winterbourne CC, Hampton MB (2010). Measuring the Redox State of Cellular Peroxiredoxins by Immunoblotting.. Meth Enzymol.

[28] Vickers MH, Breier BH, Cutfield WS, Hofman PL, Gluckman PD (2000). Fetal Origins of Hyperphagia, Obesity, and Hypertension and Postnatal Amplification by Hypercaloric Nutrition.. Am J Physiol Endocrinol Metab.

[29] da Silva Faria T, da Fonte Ramos C, Sampaio FJ (2004). Puberty Onset in the Female Offspring of Rats Submitted to Protein or Energy Restricted Diet During Lactation.. J Nutr Biochem.

[30] Leonhardt M, Lesage J, Croix D, Dutriez-Casteloot I, Beauvillain JC (2003). Effects of Perinatal Maternal Food Restriction on Pituitary-Gonadal Axis and Plasma Leptin Level in Rat Pup at Birth and Weaning and on Timing of Puberty.. Biology of Reproduction.

[31] Ozanne SE, Hales CN (2004). Lifespan: Catch-up Growth and Obesity in Male Mice.. Nature.

[32] Ozanne SE, Nicholas Hales C (2005). Poor Fetal Growth Followed by Rapid Postnatal Catch-up Growth Leads to Premature Death.. Mech Ageing Dev.

[33] Picton HM (2001). Activation of Follicle Development: The Primordial Follicle.. Theriogenology.

[34] Keisler DH, Lucy MC (1996). Perception and Interpretation of the Effects of Undernutrition on Reproduction.. J Anim Sci.

[35] Dong J, Albertini DF, Nishimori K, Kumar TR, Lu N (1996). Growth Differentiation Factor-9 Is Required During Early Ovarian Folliculogenesis.. Nature.

[36] Vitt UA, McGee EA, Hayashi M, Hsueh AJ (2000). In Vivo Treatment with GDF-9 Stimulates Primordial and Primary Follicle Progression and Theca Cell Marker Cyp17 in Ovaries of Immature Rats.. Endocrinology.

[37] Nilsson EE, Skinner MK (2002). Growth and Differentiation Factor-9 Stimulates Progression of Early Primary but Not Primordial Rat Ovarian Follicle Development.. Biol Reprod.

[38] Orisaka M, Orisaka S, Jiang JY, Craig J, Wang Y (2006). Growth Differentiation Factor 9 Is Antiapoptotic During Follicular Development from Preantral to Early Antral Stage.. Mol Endocrinol.

[39] Rosenfeld CS, Wagner JS, Roberts RM, Lubahn DB (2001). Intraovarian Actions of Oestrogen.. Reproduction.

[40] Clegg DJ, Brown LM, Woods SC, Benoit SC (2006). Gonadal Hormones Determine Sensitivity to Central Leptin and Insulin.. Diabetes.

[41] Ricci AG, Di Yorio MP, Faletti AG (2006). Inhibitory Effect of Leptin on the Rat Ovary During the Ovulatory Process.. Reproduction.

[42] Roman EA, Ricci AG, Faletti AG (2005). Leptin Enhances Ovulation and Attenuates the Effects Produced by Food Restriction.. Mol Cell Endocrinol.

[43] Spicer LJ (2001). Leptin: A Possible Metabolic Signal Affecting Reproduction.. Domest Anim Endocrinol.

[44] Spicer LJ, Chamberlain CS, Francisco CC (2000). Ovarian Action of Leptin: Effects on Insulin-Like Growth Factor-I-Stimulated Function of Granulosa and Thecal Cells.. Endocrine.

[45] Kumar TR, Wang Y, Lu N, Matzuk MM (1997). Follicle Stimulating Hormone Is Required for Ovarian Follicle Maturation but Not Male Fertility.. Nat Genet.

[46] Agarwal A, Gupta S, Sharma RK (2005). Role of Oxidative Stress in Female Reproduction.. Reprod Biol Endocrinol.

[47] Orrenius S, Gogvadze V, Zhivotovsky B (2007). Mitochondrial Oxidative Stress: Implications for Cell Death.. Annu Rev Pharmacol Toxicol.

[48] Kaipia A, Hsueh AJ (1997). Regulation of Ovarian Follicle Atresia.. Annu Rev Physiol.

[49] Tilly JL (1996). Apoptosis and Ovarian Function.. Rev Reprod.

[50] Tarry-Adkins JL, Chen JH, Smith NS, Jones RH, Cherif H (2009). Poor Maternal Nutrition Followed by Accelerated Postnatal Growth Leads to Telomere Shortening and Increased Markers of Cell Senescence in Rat Islets.. FASEB J.

[51] Simmons RA (2006). Developmental Origins of Diabetes: The Role of Oxidative Stress.. Free Radic Biol Med.

[52] Tarry-Adkins JL, Chen JH, Jones RH, Smith NH, Ozanne SE (2010). Poor Maternal Nutrition Leads to Alterations in Oxidative Stress, Antioxidant Defense Capacity, and Markers of Fibrosis in Rat Islets: Potential Underlying Mechanisms for Development of the Diabetic Phenotype in Later Life.. FASEB J.

[53] Suzuki T, Sugino N, Fukaya T, Sugiyama S, Uda T (1999). Superoxide Dismutase in Normal Cycling Human Ovaries: Immunohistochemical Localization and Characterization.. Fertil Steril.

[54] Jozwik M, Wolczynski S, Szamatowicz M (1999). Oxidative Stress Markers in Preovulatory Follicular Fluid in Humans.. Mol Hum Reprod.

[55] Paszkowski T, Traub AI, Robinson SY, McMaster D (1995). Selenium Dependent Glutathione Peroxidase Activity in Human Follicular Fluid.. Clin Chim Acta.

[56] Chang TS, Cho CS, Park S, Yu S, Kang SW (2004). Peroxiredoxin Iii, a Mitochondrion-Specific Peroxidase, Regulates Apoptotic Signaling by Mitochondria.. J Biol Chem.

[57] Tarin JJ (1995). Aetiology of Age-Associated Aneuploidy: A Mechanism Based on the ‘Free Radical Theory of Ageing’.. Hum Reprod.

[58] Tarin JJ (1996). Potential Effects of Age-Associated Oxidative Stress on Mammalian Oocytes/Embryos.. Mol Hum Reprod.

[59] Billig H, Furuta I, Hsueh AJ (1993). Estrogens Inhibit and Androgens Enhance Ovarian Granulosa Cell Apoptosis.. Endocrinology.

[60] Edson MA, Nagaraja AK, Matzuk MM (2009). The Mammalian Ovary from Genesis to Revelation.. Endocr Rev.

[61] Connor KL, Vickers MH, Cupido C, Sirimanne E, Sloboda DM (2010). Maternal High Fat Diet During Critical Windows of Development Alters Adrenal Cortical and Medullary Enzyme Expression in Adult Male Rat Offspring.. Journal of Developmental Origins of Health and Disease.

[62] Howie GJ, Sloboda DM, Kamal T, Vickers MH (2009). Maternal Nutritional History Predicts Obesity in Adult Offspring Independent of Postnatal Diet.. J Physiol.

[63] Matsuzawa-Nagata N, Takamura T, Ando H, Nakamura S, Kurita S (2008). Increased Oxidative Stress Precedes the Onset of High-Fat Diet-Induced Insulin Resistance and Obesity.. Metabolism.

[64] Vincent HK, Innes KE, Vincent KR (2007). Oxidative Stress and Potential Interventions to Reduce Oxidative Stress in Overweight and Obesity.. Diabetes Obes Metab.

[65] Couillard C, Ruel G, Archer WR, Pomerleau S, Bergeron J (2005). Circulating Levels of Oxidative Stress Markers and Endothelial Adhesion Molecules in Men with Abdominal Obesity.. J Clin Endocrinol Metab.

[66] Hirshfield AN, Midgley AR (1978). Morphometric Analysis of Follicular Development in the Rat.. Biol Reprod.

